# CardioNet: a manually curated database for artificial intelligence-based research on cardiovascular diseases

**DOI:** 10.1186/s12911-021-01392-2

**Published:** 2021-01-28

**Authors:** Imjin Ahn, Wonjun Na, Osung Kwon, Dong Hyun Yang, Gyung-Min Park, Hansle Gwon, Hee Jun Kang, Yeon Uk Jeong, Jungsun Yoo, Yunha Kim, Tae Joon Jun, Young-Hak Kim

**Affiliations:** 1 Department of Medical Science, Asan Medical Institute of Convergence Science and Technology, Asan Medical Center, University of Ulsan College of Medicine, Seoul, Republic of Korea; 2 Division of Cardiology, Department of Internal Medicine, Eunpyeong St. Mary’s Hospital, The Catholic University of Korea, Seoul, Republic of Korea; 3 Department of Radiology, Asan Medical Center, University of Ulsan College of Medicine, Seoul, Republic of Korea; 4 Department of Cardiology, Ulsan University Hospital, University of Ulsan College of Medicine, Ulsan, Republic of Korea; 5 Big Data Research Center, Asan Institute for Life Sciences, Asan Medical Center, 88, Olumpicro 43gil, Songpagu, Seoul, 05505 Republic of Korea; 6 Division of Cardiology, Department of Internal Medicine, Asan Medical Center, University of Ulsan College of Medicine, 88, Olumpicro 43gil, Songpagu, Seoul, 05505 Republic of Korea

**Keywords:** Cardiovascular diseases, Database, Artificial intelligence, Electronic health records

## Abstract

**Background:**

Cardiovascular diseases (CVDs) are difficult to diagnose early and have risk factors that are easy to overlook. Early prediction and personalization of treatment through the use of artificial intelligence (AI) may help clinicians and patients manage CVDs more effectively. However, to apply AI approaches to CVDs data, it is necessary to establish and curate a specialized database based on electronic health records (EHRs) and include pre-processed unstructured data.

**Methods:**

To build a suitable database (CardioNet) for CVDs that can utilize AI technology, contributing to the overall care of patients with CVDs. First, we collected the anonymized records of 748,474 patients who had visited the Asan Medical Center (AMC) or Ulsan University Hospital (UUH) because of CVDs. Second, we set clinically plausible criteria to remove errors and duplication. Third, we integrated unstructured data such as readings of medical examinations with structured data sourced from EHRs to create the CardioNet. We subsequently performed natural language processing to structuralize the significant variables associated with CVDs because most results of the principal CVD-related medical examinations are free-text readings. Additionally, to ensure interoperability for convergent multi-center research, we standardized the data using several codes that correspond to the common data model. Finally, we created the descriptive table (i.e., dictionary of the CardioNet) to simplify access and utilization of data for clinicians and engineers and continuously validated the data to ensure reliability.

**Results:**

CardioNet is a comprehensive database that can serve as a training set for AI models and assist in all aspects of clinical management of CVDs. It comprises information extracted from EHRs and results of readings of CVD-related digital tests. It consists of 27 tables, a code-master table, and a descriptive table.

**Conclusions:**

CardioNet database specialized in CVDs was established, with continuing data collection. We are actively supporting multi-center research, which may require further data processing, depending on the subject of the study. CardioNet will serve as the fundamental database for future CVD-related research projects.

## Background

Cardiovascular diseases (CVDs) are disorders related to the heart and blood vessels responsible for the blood supply in the human body. According to the World Health Organization (WHO), every year, an estimated 17 million people globally die of CVDs, particularly heart attacks and strokes [1]. CVDs are often caused by environmental factors, such as obesity, smoking, drinking, and stress, or genetic factors, such as underlying disease and family history. CVDs require relatively intense management, and the morbidity of long-term complications is high. In particular, metabolic diseases such as hypertension, diabetes, and dyslipidemia are typical complications. Most of these diseases have no serious symptoms at their onset and are difficult to diagnose, making it easy to overlook the risk or severity of CVDs. Therefore, it is necessary to analyze the cardiovascular-related clinical data to identify risk factors and to develop a predictive model that can help clinicians accurately diagnose the diseases early through consideration of individual characteristics of patients.

Recent advances in artificial intelligence (AI) technology have enabled early detection of several diseases and have already shown performance that approximates or exceeds that of a physician [2–4]. Deep learning technology, such as the Convolutional Neural Network and Recurrent Neural Network, has shown great results in the analysis of raw medical image and signal data [5–8]. Conversely, the performance of AI technology using structured medical data stored in electronic health records (EHRs) does not reach the effectiveness seen in image and signal analyses [9, 10]. AI approaches likely excel in medical imaging and signal examinations because of the specialized application of these modalities in the diagnoses of distinct diseases and their use for an explicit purpose. When a disease is diagnosed or suspected, there are inherent representative signs or patterns. The advanced abilities of deep learning to analyze images and signal data rely on the ability to segment given data, identify and learn features directly from the data with minimal external manipulation, and accurately distinguish the essential and inherent features.

For the patient-specific evaluation of CVDs, a database optimized for CVDs based on EHR should be established. EHRs have no specific purpose; they collect and store basic medical information cumulatively such as records of encounters, physical measurements, diagnoses, and medications. Furthermore, unstructured data containing medical information that is relatively important for diagnosis of CVDs include image and signal examinations such as echocardiography, coronary artery computed tomography (CT), electrocardiography (ECG), and cardiac stress test. Since unstructured readings are written in text, it is difficult to use them to train the AI models. Once these unstructured data have undergone proper pre-processing, they should also be integrated into the database to allow for intensive and multi-faceted CVD-related AI research.

**Fig. 1:**
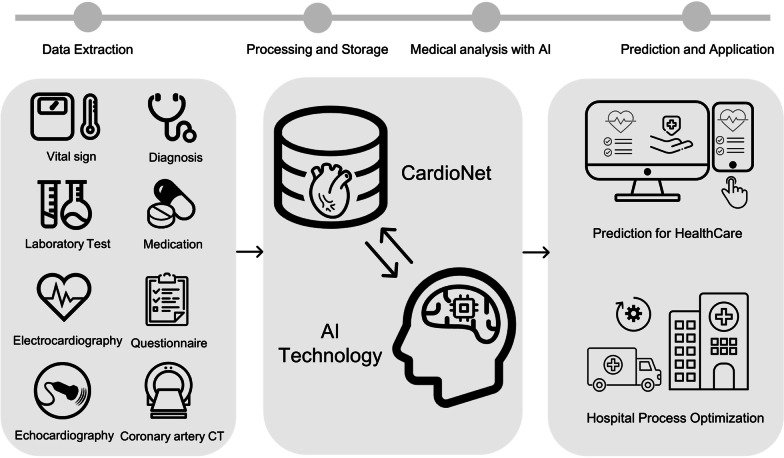
Overview of CardioNet

Constructing an advanced systematic database allows AI technology to be used efficiently to help clinicians and patients make decisions at each stage of clinical care. In particular, since CVDs require long-term and active management, it is essential for AI to utilize the patients’ characteristics, rather than existing diagnostic-based systems. The schema for the specialized cardiovascular database (CardioNet) is shown in Fig. 1.

**Fig. 2:**
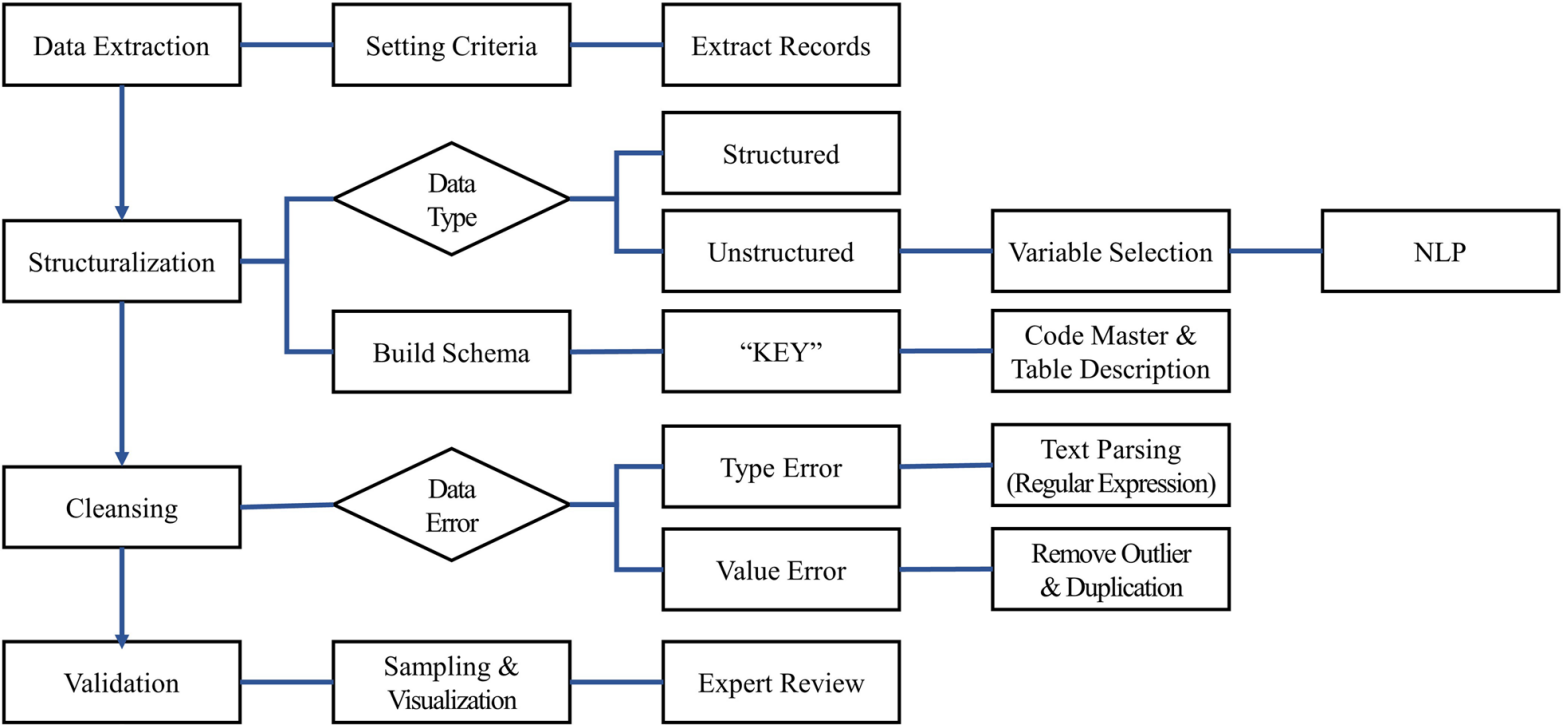
The overall process of building the CardioNet

**Fig. 3:**
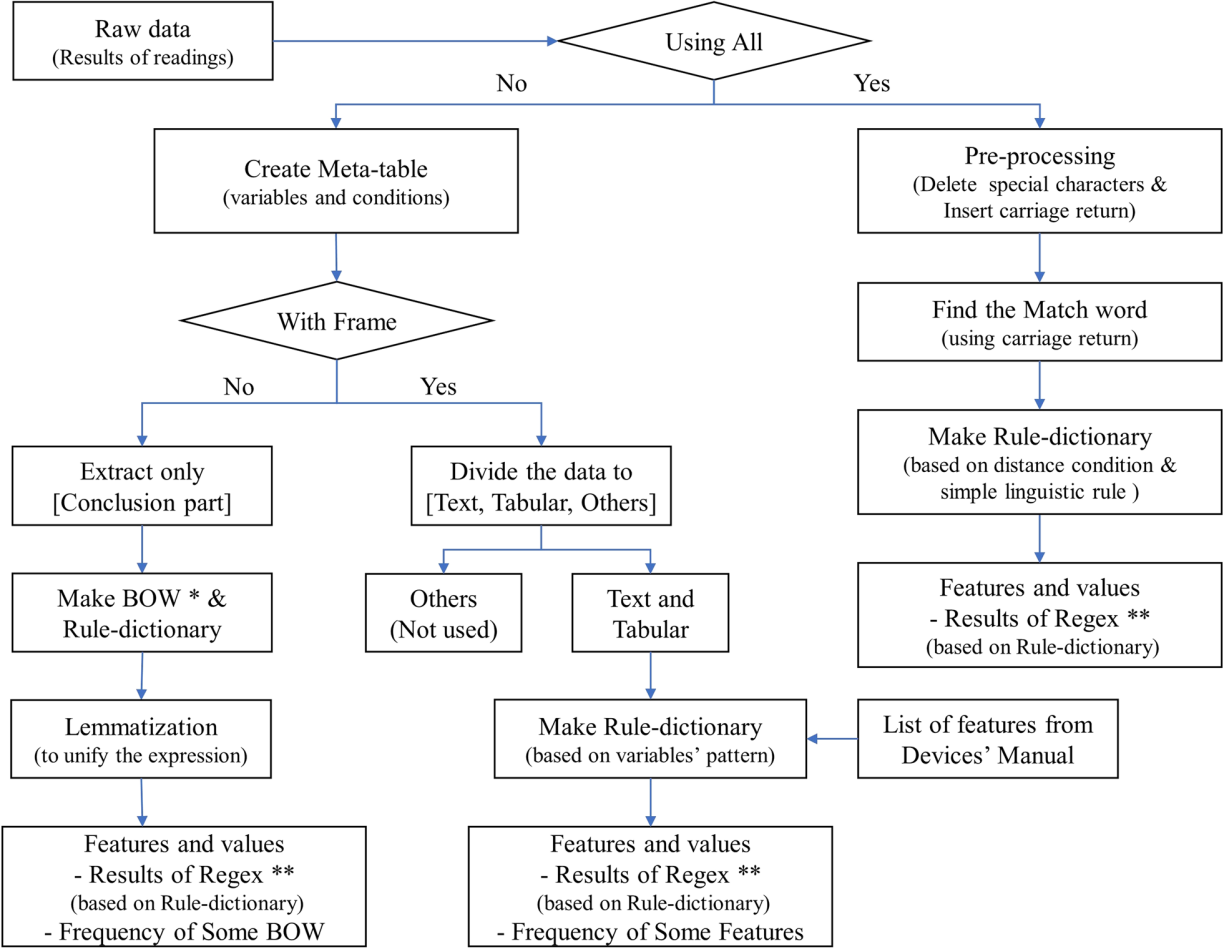
The flow chart of natural language processing. *Bag-of-words (BOW); **Regular expression (Regex)

**Fig. 4:**
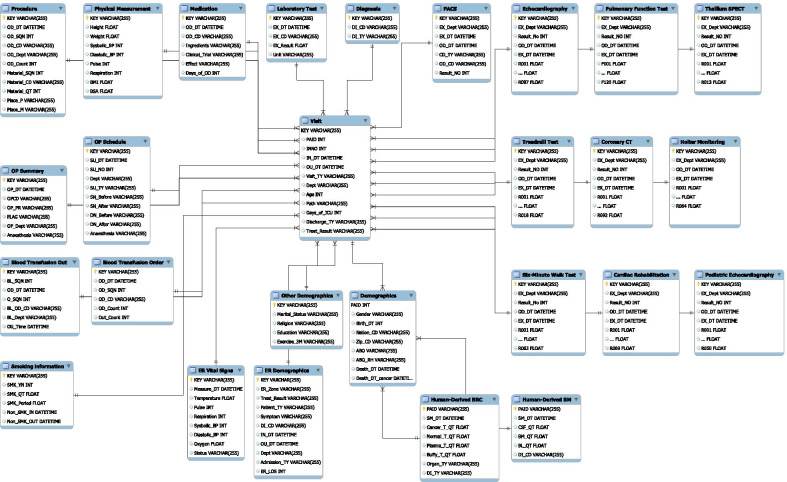
ERD of CardioNet

The purpose of this study was to build CardioNet for CVDs that will allow AI technology to be applied to clinical “big data” based on EHRs. Through this work, we expect to contribute to the discovery of risk factors and detection of their interactions, with the ultimate goal of preventing disease progression and improving treatment planning by early prediction of the occurrence of CVD and better management of prognosis in overall care for CVDs.

The data used in this study are the records of patients who visited the Asan Medical Center (AMC) in Seoul, Korea, or Ulsan University Hospital (UUH) in Ulsan, Korea, between January 1, 2000, and December 31, 2016. Data were collected from patients diagnosed with heart disease or suspected of having heart disease at the emergency room (ER) or the Departments of Cardiology or Thoracic Surgery or have undergone CVD-related examinations such as echocardiograpy, ECG, coronary artery CT, and treadmill stress tests.

The main contribution of this work can be summarized in the steps used to establish and optimize CardioNet. First, we integrated unstructured data such as readings of medical examinations with structured data based on EHRs in this study. We built a large-scale and comprehensive clinical database that is suitable for use within diverse medical AI research. Currently, most clinical AI research is conducted as independent studies in specific areas of imaging and signals. However, patients’ clinical outcomes and data should be analyzed with an integrated approach. CardioNet is a database that contains key information about CVDs and can be used as a training data set for AI models on a number of CVD-related topics.

Second, we performed natural language processing (NLP) to structuralize the unstructured information, refining the data to allow for immediate application of AI. Specifically, since the results of CVD-related imaging or signal examinations are mostly written in free-text, NLP was needed to improve the performance of AI, which depends on the pre-processing of the data. In this process, the clinical knowledge naturally included in the data can increase reliability.

Third, we standardized the data in the CardioNet for convergent multi-center research to ensure interoperability. The structure and rules of the EHRs and the coding system are different at each hospital, making multi-institutional research difficult. Standardized code systems have been proposed and utilized for a long time, and there is a trend to take advantage of the globally integrated code system standards that unify data structures, such as the common data model (CDM). However, because the standardization system for the results of various tests, such as imaging for CVDs, is not yet included in the CDM, we created our own variables for them. It will be possible to convert the system quickly when the relevant standardization is established.

## Methods

The overall process for building CardioNet is depicted in Fig. 2. There are a total of five steps involved: data extraction, structuralization, cleansing, standardization, and validation. A detailed description of each step is as follows.

### Description of patients

#### Anonymity of data

The collection of data and data preparation received AMC and UUH institutional review board approval (IRB) with waived informed consent. It is mandatory for all researchers to protect patients’ privacy and to make sure that the data cannot be traced back. AMC has a system called “ABLE (Asan BiomedicaL Environment)”, in which only authorized researchers to review anonymous sample data and to extract data after IRB approval. Also, the de-identification and extraction process in this system are conducted through the IT service management unit of AMC’s medical information office and the honest broker of the research information unit, not the research participants. UUH’s data was similarly anonymized. The list of de-identified information in line with the health insurance portability and accountability act (HIPAA) and hospital policy is shown in Table 1.

**Table 1:** The list of De-identified data

No.	De-identified information
1	Unique identification information (resident/alien registration number, passport number)
2	Names (including Chinese characters, English name, pen name, etc.)*
3	Detailed address (detailed address below eup/myeon/dong)
4	All phone numbers (including mobile phone/home/company/fax number)
5	E-mail addresses
6	Medical record number
7	Patient registration number
8	Health insurance card number, Welfare recipient number
9	Accounts number, Credit card number
10	Certificate/License number, Student number
11	Vehicle number, registration number & serial number of various devices
12	Full-face photographs or equivalent (still photo, video, CCTV, video)
13	Identification code (member ID, employee number)
14	IP (Internet Protocol) address, Mac (Media Access Control) address
15	URLs (Universal Resource Locators)
16	Biometric identifiers: fingerprint, iris, vein, voice, handwriting, personally identifiable genetic information
17	Any other personally identifiable information (pathological number)
18	Date of birth**
19	Any other unique identifying information (military number, registration number of the individual business operator)
20	The indirect identification information contained in the information collection is also deleted in principle if it is not related to the purpose of data use.

*Including medical personnel

**“Date of birth” is not personally identifiable information, IRB approval is required if information up to “date” is required

**Table 2:** The mapping ratio of AMC’s code

Class	Total codes (*N*)	Mapped code (*N*)	Mapping ratio (%)	Remark
Diagnosis	10728	10708	99.81	SNOMED-CT
Surgery	1554	1544	99.36	SNOMED-CT
Laboratory test	705	599	84.96	LOINC*
Image pathology	247	245	99.19	SNOMED-CT
Medication	4631	4600	99.33	RxNorm
Blood transfusion	23	23	100	SNOMED-CT
Procedures and materials	386	382	98.96	SNOMED-CT

SNOMED-CT: systematized nomenclature of medicine-clinical terms

LOINC: logical observation identifiers names and codes

RxNorm: a standardized nomenclature for clinical drugs produced by the U.S. National Library of Medicine(NLM)

*Except for bacterial code

**Table 3:** Summary of CardioNet

Description	Features (*N*)	Number of records		Number of patients	
		AMC	UUH	AMC	UUH
Demographics	9	572811	175663	572811	175663
Demographics of those visiting ER	20	502055	171489	214393	72423
Vital signs of those visiting ER	13	1865348	–	185447	–
Physical measurement	14	46768559	5485196	511061	130361
Visits	23	18967703	8935764	571163	172169
Diagnosis	13	28328713	8089345	553031	174403
Schedule of operation	12	434085	–	245159	–
Summary of operation	14	3404439	88760	348939	52852
Six-minute walk test	74	32158	1210	8871	665
Coronary artery CT	97	97585	–	79046	–
Thallium SPECT	26	198711	–	156615	–
Echocardiography	112	726187	178386	428004	110626
Holter monitoring test	75	66366	21035	46636	15135
Pulmonary function test	135	4634091	63593	265817	38933
PACS	12	12410683	4490786	551280	169801
Pediatric echocardiography	63	4017	–	1720	–
Cardiac rehabilitation	80	2912	–	1990	–
Treadmill test	29	110094	31741	68203	25979
Laboratory test	7	344908032	143847546	489278	175663
Medication	26	129804022	57639868	500444	162750
Procedures and materials	21	105739326	13201735	417407	136128
Order of blood transfusion	10	1090115	219804	192169	43814
Result of blood transfusion	11	2764232	625574	100215	28621
Human-derived materials	13	46760	–	43412	–
Human-derived bonemarrow	13	5757	–	2983	–
Patient history	10	673143	–	307681	–
Smoking information	12	608441	–	280492	–

**Table 4:** Demographics

	AMC (*N* = 572811)	UUH (*N* = 175663)	Total (*N* = 748474)
Gender ([F,M])	[257160, 315651]	[79988, 95675]	[337148, 411315]
Age (Year)	56.32 $\pm$ 14.72	52.11 $\pm$ 18.09	55.78 $\pm$ 15.20
Systolic blood pressure (mmHg)*	123.06 $\pm$ 12.61	129.05 $\pm$ 13.38	124.14 $\pm$ 12.95
Diastolic blood pressure (mmHg)*	74.29 $\pm$ 7.94	75.96 $\pm$ 9.07	74.59 $\pm$ 8.18
BMI (kg/m$^{2}$)**	24.11 $\pm$ 3.50	24.04 $\pm$ 3.55	24.100 $\pm$ 3.513
CV/CS Encounter(*N*) ***			
0	250160	14925	265085
1	68037	19489	87526
2	78406	19101	97507
$\ge$ 3	174560	118654	293214
Test (*N*(%))			
Echocardiography	428004 (74.71%)	110626 (62.97%)	538630 (71.96%)
Pulmonary function	265817 (46.40%)	38933 (22.16%)	304750 (40.71%)
Thallium SPECT	156615 (27.34%)	–	156615 (20.92%)
Treadmill	68203 (11.90%)	25979 (14.78%)	94182 (12.58%)
CT	79064 (13.80%)	–	79064 (10.56%)
Holter monitoring	46636 (8.14%)	15135 (8.61%)	61771 (8.25%)
Six-minute walk test	8871 (1.54%)	665 (0.37%)	9536 (1.27%)
Cardiac rehabilitation	1990 (0.34%)	–	1990 (0.26%)
Pediatric echocardiography	1720 (0.30%)	–	1720 (0.22%)

*N of Blood Pressure: AMC = 461693, UUH = 101438

**N of BMI : AMC = 457621, UUH = 77171

***N of Visits total : AMC = 571163, UUH = 172169

**Table 5:** Number of visits to the departments of Cardiology or Thoracic surgery

	AMC (*N* = 321003)	UUH (*N* = 157244)	Total (*N* = 478247)
Age (Year)	59.85 $\pm$ 13.21	57.28$\pm$ 15.00	58.80 $\pm$14.03
Outpatients	2548245	1854432	4402677
Inpatients	134846	71012	205858
ER	86429	–	86429

**Table 6:** The number of patients with major diseases

Diagnosis	AMC	UUH	Total
	(*N* = 357910)	(*N* = 87877)	(*N* = 445787)
Hypertension	200109 (55.91%)	37886 (43.11%)	237995 (53.38%)
Pain in throat and chest	142567 (39.83%)	38690 (44.02%)	181257 (40.66%)
Diabetes mellitus	112381 (31.39%)	30236 (34.40%)	142617 (31.99%)
Angina pectoris	61789 (17.26%)	9694 (11.03%)	71483 (16.03%)
Ischaemic heart disease	47836 (13.36%)	5847 (6.65%)	53683 (12.04%)
Cerebral infarction	24752 (6.91%)	8958 (10.19%)	33710 (7.56%)
Heart failure	15345 (4.28%)	4825 (5.49%)	20170 (4.52%)
Acute myocardial infarction	10543 (2.94%)	3853 (4.38%)	14396 (3.22%)
Cardiac arrest	1213 (0.003%)	1196 (0.013%)	2409 (0.005%)

**Table 7:** Percentage of patients with laboratory results

Laboratory test	AMC(%)	UUH(%)	Total(%)
Creatinine	83.64	91.24	85.42
Cholesterol	83.60	93.25	85.86
ALT	83.53	92.21	85.57
AST	83.53	92.26	85.58
Bilirubin (total)	83.03	91.29	84.97
Albumin	83.02	91.34	84.97
Protein	83.01	91.30	84.96
Glucose	83.00	90.47	84.75
ALP	82.97	91.25	84.91
Hb	82.88	92.51	85.14
Platelet	82.88	92.48	85.13
Calcium	82.77	87.37	83.84
Uric acid	82.68	89.44	84.27
Potassium	80.54	87.91	82.27
Sodium	80.51	87.95	82.25
BUN	75.36	91.08	79.05
Chloride	65.94	87.42	70.98
CO2 (total)	65.89	83.48	70.02
Phosphorus	62.40	87.40	68.27
Triglyceride	53.61	37.55	49.84
HDL-Cholesterol	52.96	37.17	49.26
LDL-Cholesterol	44.09	31.45	41.12
CRP (quantity)	42.94	67.49	48.71
ESR	42.79	42.70	42.77
Hb A1c	38.66	32.00	37.09
CK	27.76	43.13	31.37
Troponin-I	25.48	11.62	22.23
hsCRP	17.64	14.67	16.94

**Table 8:** Prime features in echocardiography

Description	AMC (*N* = 428,004)	UUH (*N*=110,626)
LVESD	30.25 $\pm$ 6.43	30.30 $\pm$ 5.76
LVEDD	47.77 $\pm$ 8.74	47.20 $\pm$ 5.57
LVPWES	13.93 $\pm$ 2.90	14.32 $\pm$ 2.25
LVPWED	9.02 $\pm$ 1.87	9.25 $\pm$ 1.55
LVIVSES	13.14 $\pm$ 2.88	13.42 $\pm$ 2.26
LVIVSED	9.09 $\pm$ 2.01	9.52 $\pm$ 1.74
LAd	37.39 $\pm$ 8.72	36.04 $\pm$ 6.23
LVESV	35.99 $\pm$ 18.94	36.83 $\pm$ 23.61
LVEDV	88.97 $\pm$ 32.39	80.82 $\pm$ 33.07
E/A ratio	0.93 $\pm$ 0.52	0.44 $\pm$ 0.54
E/E ratio	8.52 $\pm$ 7.18	9.64 $\pm$ 3.63
LVEF	58.83 $\pm$ 11.93	62.18 $\pm$ 8.47
LV mass	163.78 $\pm$ 57.80	149.31 $\pm$ 45.74

LV: Left Ventricular, LVESD: LV dimension in end-systole, LVEDD: LV dimension in end-diastole, TR: Tricuspid regurgitation, LVPWES: LV posterior wall thickness in end-systole, LVPWED: LV posterior wall thickness in end-diastole, LVIVSES: LV interventricular septum thickness in end-systole, LVIVSED: LV interventricular septum thickness in end-diastole, LAd: Left atrial diameter, LVESV: LV volume in end-systole, LVEDV: LV volume in end-diastole, LVEF: LV ejection fraction

#### Details of data

Data related to all patients who had visited AMC or UUH for CVD or related complications were collected for this study. We obtained the anonymized records of 748,474 patients who had visited AMC or UUH, from January 1, 2000, to December 31, 2016. Data were obtained from 572,811 patients seen at AMC and 175,663 patients seen at UUH within the same time period. Because the depth of information stored and retained by each hospital is different, the variables were based on the relatively detailed AMC records. In order to prepare to build the CardioNet, we set the following specific criteria for inclusion of patient data:

- Patients who had visited the Departments of Cardiology or Thoracic Surgery.
- Patients who had visited the ER and were assigned International Classification of Diseases, 10th version (ICD-10) codes related to CVDs.
- The codes I00-I99 were related to the diseases of the circulatory system, while R00-R03, R06, R068, R073, and R074 were related to symptoms and signs involving the circulatory and respiratory systems.
- Patients who had undergone coronary artery CT as part of their health screening procedures.
- Patients who had undergone one of the following clinical examinations: thallium single-photon emission computed tomography (SPECT), 2D-echocardiography, treadmill test, and Holter monitoring test.

### Data extracted

We obtained data from the EHRs, order communication system, and picture archiving and communication system (PACS). These medical record systems contain information such as patient demographics, vital signs, encounters (e.g., inpatient/outpatient visits, ER visits, and health screening), physical measurements, diagnosis, surgery (order, schedule, and summary), digital medical tests (CT, magnetic resonance imaging [MRI], X-ray, coronary angiography, echocardiography, Holter monitoring, treadmill tests, etc.), laboratory tests (pathology order and result), medication (order, prescription, and history), procedures (order and materials), blood transfusion (order and result), human-derived materials, patient history questionnaire (personal and family medical history, lifestyle and habits), and billing and claim history. By using the service of a pre-built clinical data warehouse, we extracted structured and unstructured data separately, with all data undergoing the process of de-identification.

Data extracted for the establishment of CardioNet included the following:

- Demographics: date of birth, sex, national code, address, blood type (ABO, RH), death date, and death date of a cancer patient (one row per patient).
- Vital signs: measurement time and date, reason for absence of measurement, body temperature, blood pressure, respiratory rate, pulse, oxygen saturation, and consciousness status (one row for each patient seen in the ER).
- Physical information: age, height, weight, blood pressure, pulse, respiratory rate, body mass index (BMI), body surface area, and measurement date (one row per encounter).
- Visits: date of visit, date of admission and discharge, type of visit, medical department, hospitalization, duration of stay in the intensive care unit, type of discharge, and the result of treatment (one row per encounter).
- Diagnosis: date of diagnosis, type of visit, medical department, and ICD-10th code (one row per encounter).
- Surgery: date of admission and discharge, date of surgery or treatment, sequential number of surgery, surgery type (before/after the surgery), diagnosis (before/after the surgery), surgery category, surgical department, and method and time of anesthesia (one row per encounter).
- Digital tests: date of visit, age, department of examination, code of examination, date of order, and reports or readings of the result. (one row per test).
- Laboratory test result: code of pathology examination, number of work, test result, and unit of result (one row per test).
- Medication: medical department, type of visit, date of prescription, code of prescription, active ingredient in medication, indication, category of medicinal effect, and duration of treatment (one row per encounter).
- Procedure: medical department, date of order, code of order, time of order, material code, capacity of materials, and place of patient and materials (one row per procedure).
- Blood transfusion: date of order, code of order, ordering department, sequential number of blood, quantity of prescribed/released blood, and time released (one row per order).
- Human-derived material: date of extraction, code of diagnosis, name of diagnosis, tissue sample description (status and amount of cancer/normal, plasma/buffy coat, type of organ), and information on bone marrow (status and amount of cerebrospinal fluid/bone marrow/blood stored) (one row per patient).
- Patient history: marital status, religion, education, exercise habits over the last three months, lifestyle and habits information (e.g., alcohol, smoking), and personal and family medical history (one row per encounter).

### Data processing

Before pre-processing, a Primary Key was generated to connect the data. All data, except demographics and information on human-derived materials, include the patient ID (PAID) and the patient’s encounter number (INNO) columns. KEY column was created by concatenating the PAID and INNO to each table to connect all data. Demographics and information on human-derived materials are unique contents that are modified only when the information changes. For this reason, they do not have INNOs, but are linked to other data with PAID. As an example, a patient with the PAID of 100 who visits the hospital for the first time would have the KEY of ‘100_1’.). In the existing in-hospital medical record system, it was inconvenient to extract all data satisfying specific conditions. We were able to extract data easily and quickly by connecting all data through the KEY.

#### Structured data

Most of the extracted structured data were quantitative in nature with simple and formal structures, making the pre-processing relatively simple. However, some data required further processing through the removal of data errors and outliers based on clinical knowledge, such as clarifying the meaning of each result and comparing the value to the normal range. Selected aspects of this processing are described below.

*Physical information and vital signs* Physical information including body measurements, vital signs such as height, weight, blood pressure, pulse, and respiratory rate, are continuous variables, so we identified the distribution of each and removed the implausible data judged to be errors and outliers from a clinical point of view. The following criteria were used:

- Systolic blood pressure, diastolic blood pressure between 0 and 300 mmHg
- The respiratory rate is between 0 and 100 breaths per minute.
- The pulse rate is between 0 and 300 beats per minute.
- The body temperature is between 0 and 50 $^\circ$C.
- To determine the range of the plausible body weight and height, we divided the data into three groups: patients younger than 12 months, younger than 20 years, and older than 20 years. We manually calculated the mean of values and $\pm$ 3 standard deviations for each group.

*Laboratory test results* Laboratory test results have variables such as the date of examination, code of order and examination, and results. In total, there are 8,088 examination codes, which are related to clinical pathology and nuclear medicine, with approximately 1.1 billion records associated with 748,474 patients. However, there are many overlapping records, because different prescription codes can be used for the same test and result. Since the examination result is more important than the path of the prescription, duplicate data were removed based on examination results. Moreover, each examination has a process in which a person enters the result directly, potentially introducing human errors, so secondary data cleansing was performed considering the types of value (integer, float, categorical, etc.) that should be the result of each examination. As a result, a total of 480 million records with 1,335 examination codes are included in the laboratory test results.

*Patient history questionnaire* At the time of admission, the health-related history is obtained, including information such as details of hospitalization, vital signs, personal and family medical history, past diagnoses, clinical symptoms, and lifestyle. Information on smoking, including status, period, amount per day, quit-smoking period, and quit-smoking education training status, was extracted from the questionnaire data. Based on a review of pack-year distribution in the patient population, the range was found to be generally between 0 and 500, which suggests that the data can be considered relatively reliable. With regard to the disease history, we identified some diseases that could be considered as complications of CVDs, such as diabetes, hypertension, tuberculosis, and hepatitis. We obtained patient history data, including marital status, exercise, religion, and education, along with smoking history.

#### Unstructured data

The major CVD-related examinations are echocardiography, Holter monitoring, ECG, thallium SPECT, and coronary artery CT. The majority of results from these tests are readings formatted as free-text, recorded as a mixture of Korean, English, numerical values, and special characters. Despite the presence of numerous errors and nulls introduced by the fact that most of the entries were handwritten, there are significant variables associated with CVDs. Therefore, the process of data formalization through NLP is essential.

We performed NLP on the readings of major digital examinations related to CVDs, deriving variables with high clinical importance that can be directly applied to AI models. The basic method of NLP applied to unstructured data can be described in three steps: First, we created a meta-table consisting of the main variables and conditions of extraction by the clinician. Second, we divided the readings into three frames: text, tabular, and others, and defined the extraction rules for each frame. We took into consideration the structure of the original data and the location of variables set in the meta-table and defined rules using a variety of operators and regular expressions. Third, the new tables were built by extracting the keywords and features from the original data. The values of keywords were based on rules defined in the previous step. This approach was used to process the six-minute walk test, pulmonary function test, cardiac rehabilitation, pediatric echocardiography, and treadmill tests. A simplified NLP flow chart is depicted in Figure 3

Additionally, information extracted from PACS contains records of CVD-related tests performed in over 96% of patients, including outcomes of cardiac examinations, imaging, interventional procedures, and arrhythmia assessments. We performed NLP to extract the examination codes to help classify and layer data in other tables. The specific methods used for echocardiography, Holter monitoring, thallium SPECT, and coronary CT are as follows:

*Echocardiography* Compared with other digital tests, echocardiography is the most common test performed in patients with CVDs, with a total of 538,630 patients (71% of the entire CardioNet population) undergoing this test. Since the results of echocardiography are sentences in free-text form without a frame, this information was processed with specific rules for extracting values.

First, the “Conclusion” part of the reading, which includes a summary of important values of test results and the clinicians’ interpretation, is set to the extraction range. As primary verification, we investigated all words in the “Conclusion” section and corrected typographical and grammatical errors. Second, we tokenized the words and created the bag-of-words (BOW) that contain the keywords and their frequencies [11].

Approximately 9,000 keywords were identified, appearing a total of 3.4 million times. We subsequently created a rule-dictionary to consistently replace errors with the correct words, since it is not always possible to scrutinize all the data. Third, as the secondary verification, the lemmatization, which is a type of normalization, was conducted. English words differ in terms of morphology depending on parts of speech or tense, so we extracted the stem of the word (the core part containing the meaning) and unified the expression, allowing it to be recognized as the same keyword. Since the meaning of the word may vary depending on context and/or affix, we modified the keywords once more following a full investigation. About 9,000 keywords were reduced to about 2,500. Additionally, because of bias and range of pre-knowledge, cross-checking between the engineer and clinician was repeated twice to improve the completeness and accuracy of the rule dictionary. Based on the clinician’s opinion and review of CVD-related research, we selected approximately 100 meaningful features among the extracted keywords and created an echocardiography table by defining patterns and extracting them into binary or continuous values.

*Holter monitoring* Holter monitoring is the examination performed using electrodes and a recording device to track the heart’s rhythm for a period of 24 to 72 hours. Although a total of 61,771 patients had undergone Holter monitoring (less than the number of patients subjected to other tests), Holter monitoring test is essential for patients with irregular cardiac rhythm. Holter monitoring tends to be more regular than other examinations because the readings are automatically generated by the test equipment. Since AMC is using the General Electric equipment, we were able to obtain a list of variables that appear in the records from the equipment manual. Meaningful keywords were subsequently selected through interpretation by the clinicians, with appearance and frequency expressed using an approach similar to that used with echocardiography data.

*Thallium SPECT* Thallium SPECT is the examination used to diagnose coronary diseases and to verify the survival of the myocardium. A total of 156,615 patients underwent thallium SPECT at AMC. In previous research, the values of summed stress score, summed rest score, and summed difference score derived from the thallium SPECT equipment were used. However, these values often do not match those in the patient records. We, therefore, conducted NLP by considering the perspectives of the clinical field. As with other modalities described above, rules were defined to find the required information, including the output from the device, and variables and values were extracted to create the table. Specifically, the position and degree at the time of stress were extracted from the readings, allowing us to deduce the position and extent of the cardiac problem. According to the patient’s condition, up to eight disorders were identified.

*Coronary artery CT* Coronary artery CT is another examination performed to diagnose CVDs and evaluate the presence of various cardiac conditions. A total of 79,046 patients at AMC underwent coronary CT. We modified the NLP pipeline to further increase the coverage of data. The key concept underlying the NLP method is the analysis of the text based on linguistic rules, focusing on keywords that are to be extracted. This method does not need to divide the structure of the readings into individual forms and can be performed for all sentences. We identified words on the same line by assigning a certain distance condition to the “carriage return,” as well as by using a “match word.” The keywords corresponding to stenosis degree and plague for each segment existing in the reading text were extracted. We completed the extraction of additional information, such as the type and patency status of vascular treatments in patients who had undergone stent graft surgery and details about the native coronary artery. After processing, we validated the data that had been structured and processed with input from clinicians and evaluated the reliability of the CardioNet data. We constructed multiple scenarios based on clinicians’ practical experiences, sampled the data, and visualized the frequency, data types, and other parameters.

#### Standardization

Securing interoperability is essential for collaborative research with other institutions and hospitals in CVDs, as well as other diseases. We carried out the standardization process on terms and codes used in the hospitals to build the CardioNet that corresponds to the CDM form. However, term mapping is not possible without practical familiarity with the usage of the code and terms in the actual clinical field, as well as with insight into the unique information used in the institution. It is necessary to seek input from highly experienced specialists in fields such as diagnosis, surgery, laboratory tests, pathology, imaging, medication, blood transfusions, and materials. Consequently, we received counsel from the AMC medical data team and modified the codes and terms into standard terms with the same meaning.

Because there are currently no common codes for digital examination in CVDs, we created independent variables based on meaningful clinician comments, which will be converted once digital test codes are defined in the CDM (e.g., in case of echocardiography, there are 97 variables and explanation), Specifically, OMOP-CDM-based local code mapping was performed because of the differences in the code systems used in each hospital [12]. The codes of diagnosis, operation, image pathology, blood transfusion, and procedure and materials were mapped based on SNOMED-CT, laboratory test results were based on LOINC, medication was based on RxNorm. The bacterial code did not proceed in accordance with the OMOP-CDM standards [13–15].

The mapping ratio of AMC’s codes based on CDM is shown in Table 2. The ratio is almost 90% or more, so it can be used immediately when expanding to other topics of study.

Using the processed and standardized data of 748,474 patients, we constructed the CardioNet schema based on the hospital EHR structure. We created a descriptive table for 27 tables sorted by category, which is intended to facilitate database usage for clinicians and engineers, allowing them to easily access and understand the data. A descriptive table is a dictionary of data variables, complementing the dictionary of word definitions and variables in the CardioNet and explaining the anatomy, physiology, and pathology of the heart. Additionally, it displays CVD-related variables, their meaning, and clinical utility. Since most variables in the table have abbreviated values (code of orders, diagnosis, examination, etc.), we created a code master table and linked these values. This makes it easy to find the meaning of words and abbreviations.

We continuously validated the data during pre-processing to further ensure the reliability of the CardioNet and verify the processed data. Furthermore, we constructed scenarios based on the clinicians’ practical experiences, sampled the data, and visualized their frequency and types.

#### CardioNet built

All data tables (except demographics and human-derived materials) have a KEY column concatenating PAID and INNO. The demographics table is the central table, consisting of 748,474 patients. The second central table is the visitation table with 743,332 patients. Demographic and human-derived material table without INNO is connected to the visit table by PAID, while all other tables are linked by KEY. Figure 4 describes the entity-relationship diagram (ERD) of CardioNet, with the full form of abbreviations in the ERD listed below.

## Results

A total of 74.8 million patients visited AMC or UUH for CVDs between January 2000 and December 2016. CardioNet is a comprehensive database intended to support the development of predictive models of CVDs and future multi-center convergent research. It comprises information that can be extracted from EHRs and has undergone structuralization and standardization by the processing of the readings of CVD-related digital tests by NLP. CardioNet contains a total of 27 tables, a code-master table, and a descriptive table.

### Summary of CardioNet

Table 3 summarizes the tables of CardioNet, providing a description of individual tables, number of features, and the number of records and patients included from each hospital.

### Demographics

A total of 572,811 patients visited AMC and 175,663 patients visited UUH. Table 4 depicts the demographics of the two hospitals, including the physical measurements and the number of patients who have undergone CVD-related digital examinations. Approximately 45% of patients were women, with an average age of 55.78 years at the time of the initial encounter. Body measurements, such as weight and height, and vital signs, such as blood pressure, are not consistently performed at each visit. As shown in Table 4, the average valid value was calculated for each patient (i.e., 563,131 patients and 543,792 patients have valid blood pressure and BMI values calculated). Blood pressure data are available for 75.23% of patients, with the average systolic blood pressure determined to be higher than 120 mmHg and therefore above the normal range, while average diastolic blood pressure was below 80 mmHg.

The WHO Asia-Pacific region and the Korean Obesity Association standards deem individuals as overweight when their BMI is 23 kg/m$^{2}$ or higher and obese when BMI is 25 kg/m$^{2}$ or higher. According to these criteria, the average BMI values show the majority of the patients to be overweight [16]. Additionally, 63.89% of patients visited the department of cardiology or thoracic surgery more than once, with 39.17% patients registering more than three visits. Patients with CVD-related diseases continued to visit the hospital. In processing the digital medical tests, duplicates were removed for each patient, and the number of cases examined more than once was determined. This analysis demonstrated that 71.96% of the patients underwent echocardiography.

### Visits

The total number of visits to the Departments of Cardiology or Thoracic Surgery is presented in Table 5, which shows the number of rows related to visits to each department. A total of 428,247 patients visited the departments more than once, accounting for 57.21% of the total. The total number of visits by these patients to both hospitals is approximately 4.69 million, accounting for 16.82% of the total number of visits (27.9 million). As shown in Table 5, the average age of these patients was 58.8 years, which is 3.01 years higher than the average age of the entire patient population. Outpatient visits comprised 92% of all visits, inpatient visits accounted for 4.86%, and approximately 3% corresponded to ER visits (with only AMC ER data considered).

### Diagnosis

Table 6 describes the number of patients diagnosed with nine major CVDs and complications. Because a single patient can be diagnosed with multiple diseases and diagnosis records are taken at each visit (Table 6), duplicates were removed and each patient’s unique diagnostic names were counted. A total of 445,787 patients (59.55%) were identified with major CVDs. Hypertension was diagnosed most frequently (31.79% of the entire CardioNet population), followed by throat and chest pain, diabetes mellitus (including types 1 and 2, malnutrition-related, unspecified, etc.), angina pectoris, chronic ischemic heart diseases, cerebral infarction, heart failure, acute myocardial infarction, and cardiac arrest. Considering that the average of the number of diseases for each patient diagnosed with the major CVDs was 1.82 (standard deviation 1.2) and that there are patients with up to nine CVDs, a number of patients were found to exhibit comorbidities and complications.

### Laboratory results

The laboratory test table contains the results of 1,335 diagnostic tests, with a total of 480 million rows of data from 664,941 patients (89% of the total population). In some cases, a single patient may undergo multiple tests during the day or the same tests several times a day. Table 7 presents the percentage of patients who have undergone each laboratory test sorted by the number of patients seen at AMC. Excluding duplicate entries, results of the following laboratory tests were found for at least half of all patients: creatinine, cholesterol, alanine transaminase, aspartate transaminase, bilirubin (total), albumin, protein, glucose, alkaline phosphatase, hemoglobin, platelets, calcium, uric acid, potassium, sodium, blood urea nitrogen, chloride, CO$_{2}$ (total), and phosphorus. Results of other tests were available for at least 15% of the patients, including triglycerides, high density lipoprotein-cholesterol, low density lipoprotein-cholesterol, C-reactive protein (quantity), erythrocyte sedimentation rate, hemoglobin A1c, creatine kinase, troponin-1, and high-sensitivity C-reactive protein.

### Echocardiography

Echocardiography accounted for the largest number of medical digital examinations undergone by patients (71.96%). Echocardiography readings were converted into 112 variables, with the basic statistics of 19 major variables reflecting clinicians’ opinion shown in Table 8. The descriptive table includes the meaning and clinical interpretation of each variable. As an example, the left ventricle (LV) dimension in systole refers to the inner diameter of the LV measured during systole, with the expansion of the LV indicated when the value in a male patient exceeds 42 mm.

## Discussions

The EHRs are easily accessible in hospitals and contain important clinical information. EHR data were found to be less useful in AI studies, compared to data from imaging modalities such as CT or MRI. We summarized the insights and expectations of building CardioNet along with its limitations.

First, in the AMC, there is cloud environment, which is an infrastructure for research with researchers inside and outside the hospital. This cloud system aims to implement a shared database and collaboration for multi-center medical AI research. Also, it is a hybrid cloud that can use a public cloud (e.g., AWS, MS Azure) based on a private cloud. Therefore, those who want to do research with CardioNet, need to register as a joint research team to the IRB according to hospital policy. Currently using CardioNet, researchers outside the AMC who have access to cloud are conducting various studies.

Second, it is difficult to directly use EHR data extracted from a hospital system to generate meaningful results with AI research. This reflects the fact that EHRs include numerous unstructured free-text entries. These free-text readings contain important clinical insights made by clinicians as part of patients’ treatment. In setting up AI research, it is necessary to decide how to handle free-text readings. The simplest way is to process the free-text notes as sentences and infer the meaning between words, as in the NLP field study [17–19]. This approach is in line with a number of existing studies that derive useful meaning by analyzing the medical articles themselves by NLP [20]. However, the biggest problem with this method is that English in free-text readings differs between clinicians, hospitals, and countries, unlike the English used in scientific communication within research articles. In different countries, these readings can be mixtures of the official national language and English. Therefore, the use of free-text readings in AI research requires significant support from clinicians. Clinicians alone can understand free-text readings, with specialized expertise in relevant topic needed for accurate interpretation.

We worked with clinicians to find valid patterns of readings, and performed rule-based NLP to convert results into variables and values. We considered various ways to apply NLP to free-text readings, but in most cases required a lot of human labor. Therefore, we are working on more efficient automatic NLP research that can be applied regardless of locality.

Also, in the process of performing NLP, we created a descriptive table (i.e., dictionary of CardioNet) that describes clinically valid variables. Although several cardiologists at AMC and UUH participated and reviewed this study, some extracted variables can be subjective compared to the numerical values. Therefore, we plan to strengthen the descriptive table by sharing it with the Korean Cardiology Association to collect the opinions of other cardiologists.

Third, in building CardioNet, we were assisted by numerous cardiologists, with most input involving standardization of the free-text readings. This is a common issue in building an EHR-based database for AI research in all types of diseases, including CVD. The current CDM for multi-center clinical research is not suitable for AI research. CDM is a good example of the standardization of deidentified patient data, with a number of hospitals building CDM-based datasets for clinical research. However, unlike clinical studies that focus on patient events, the important feature of EHR in AI research is the time-series data. The performance of AI-based predictive research based on EHRs is determined by how well the changes in the patient’s state over time are embedded in the training data. Therefore, it is necessary to develop a CDM for clinical AI research that standardizes patient events over time.

Finally, we are preparing a number of AI-based studies including automatic NLP as future work using CardioNet. Recently, deep learning technology in medical images is developing with results that exceed experts, but it is also necessary to apply AI to data linking raw images and EHR. Since the honest broker in the de-identification process has a key, the CardioNet and image and signal raw data in PACS and hospital local databases can be connected. This connection is essential for the realization of patient-specific medical care, and we are carrying out related research. As a result, we are in the process of developing and characterizing an AI model that can perform CVD risk prediction according to the characteristics of CVD, where prevention is more important than diagnosis.

## Conclusions

We built a large-scale and integrated CardioNet database to apply AI technology in CVDs for the detection of risk factors, development of predictive models for early diagnosis, and improving the care of patients. First, we obtained the EHR data with approval from the IRB of AMC and UUH. Second, we processed structured and unstructured data appropriately using medical expertise to generate data that can be directly applied to the AI model. Finally, we standardized and validated the data in CardioNet to allow multi-centered research. CardioNet can contribute to the early prediction of cardiac problems and promote further CVD-related research.

## Data Availability

The datasets analysed during the current study are not publicly available due to institutional policy but are available from the corresponding author on reasonable request.

## Abbreviations

EHR: Electronic health record
CT: Computed tomography
MRI: Magnetic resonance imaging
SPECT: Single-photon emission computed tomography
CAG: Coronary angiogram
DT: Date time
IN: Hospital in
OU: Hospital out
TY: Type NO: Number
DI: Diagnosis
DN: Diagnosis name
OD: Order
EX: Examination
CD: Code
Dept: Department
SU: Surgery
SN: Surgery name
SQN: Sequential number
QT: Quantity
BL: Blood
SM: Sampling
CSF: Cerebrospinal fluid
BM: Bone marrow
SMK: Smoking
